# Clinical utility of cardiovascular magnetic resonance in patients with cardiac rhythm management devices

**DOI:** 10.1186/1532-429X-18-S1-O124

**Published:** 2016-01-27

**Authors:** Nikhil Jariwala, Lara Bakhos, Jeffrey R Winterfield, Mark Rabbat, Mushabbar A Syed

**Affiliations:** grid.164971.c0000000109456408Cardiology, Loyola University, Chicago, IL USA

## Background

Selected patients with cardiac rhythm management devices (CRMD) can safely undergo cardiovascular magnetic resonance (CMR). However, susceptibility artifacts from CRMD generator and leads can affect image quality, limiting the information provided by CMR. We sought to evaluate whether CMR of CRMD patients can provide clinically useful information that includes answering the clinical question, providing new findings, new diagnoses, or change in clinical management.

## Methods

We studied 89 patients with CRMD who were evaluated for CMR from November 2012 through June 2015. Eighteen patients were excluded for various MRI contraindications. The remaining 71 patients completed the scan without complications. All CRMD were interrogated prior to the scan. For pacemaker dependent patients, asynchronous pacing was used during CMR. Device therapies were turned off for implantable cardioverter defibrillator (ICD) patients. CMR images were acquired on a 1.5 Tesla (Siemens Aera) scanner using standard sequences including single shot black blood imaging, cine with SSFP or FLASH, first pass perfusion, and late gadolinium enhancement (LGE). During the scan, patients were monitored with continuous vitals, ECG, oximetry, and voice contact. All CRMD were re-interrogated after the scan and original settings were restored. Images were evaluated using CMR^42^ (Circle Cardiovascular Imaging) software.

## Results

Baseline characteristics are listed in Table 1. ICD was present in 58 (81.7%) and pacemaker in 13 (18.3%) patients. Common CMR indications included evaluation for ventricular arrhythmia substrate (n = 61), hypertrophic cardiomyopathy (HCM) (n = 6), and cardiac sarcoidosis (n = 4). Device artifact affected complete evaluation of myocardial segments in 17 patients (23.9%) on cine imaging and in 50 patients (70.4%) on LGE. CMR findings are summarized in Table 2. The most common findings were non-ischemic scar (n = 24), ischemic scar (n = 17), and combined scar (n = 6), which helped guide ventricular arrhythmia ablation. Additionally, a new diagnosis or finding was made in 14 patients (19.7%), which included cardiac sarcoidosis (n = 3) and arrhythmogenic right ventricular cardiomyopathy (n = 2). In 3 HCM patients, CMR findings guided the decision for alcohol septal ablation vs. surgical resection. Overall, the clinical question was answered in 66 patients (92.9%).

**Table 1:** Baseline Clinical Characteristics

Age (years)	59.38 (± 12.95)
Male	59 (83.0%)
Body mass index (kg/m2)	29.54 (± 6.31)
Body surface area (m2)	2.09 (± 0.22)
Coronary artery disease	25 (35.7%)
Diabetes mellitus	9 (12.9%)
Hypertension	40 (57.0%)
Hyperlipidemia	37 (52.9%)
Ischemic cardiomyopathy	16 (22.9%)
Non-ischemic cardiomyopathy	24 (34.3%)
Mixed cardiomyopathy	6 (8.6%)
Previous ventricular arrhythmia ablation	24 (34.3%)

**Table 2:** CMR Findings

Non-ischemic scar	24 (33.8%)
Ischemic scar	17 (23.9%)
Combined ischemic and non-ischemic scar	6 (8.5%)
Hypertrophic cardiomyopathy	4 (5.6%)
Cardiac sarcoidosis	3 (4.2%)
Arrhythmogenic right ventricular cardiomyopathy (ARVC)	3 (4.2%)
Left ventricular involvement of ARVC	2 (2.8%)
Rejected cardiac sarcoidosis	1 (1.4%)
Rejected ARVC	1 (1.4%)
Accessory pulmonary vein	1 (1.4%)
Apical thrombus	1 (1.4%)
Pericardial lipomatosis	1 (1.4%)
Right ventricular pseudoaneurysm	1 (1.4%)
Severe aortic stenosis	1 (1.4%)
Subaortic membrane	1 (1.4%)

## Conclusions

Our results show that in carefully screened CRMD patients, CMR is safe, can answer the clinical question in vast majority of patients, and can provide new information to guide clinical management.

